# Supplementary figures and images for: Genome-Wide Identification of Solute Carrier Family 12 and Functional Characterization of Its Role in Saline–Alkaline Stress Acclimation in the Ridgetail White Shrimp Exopalaemon carinicauda
Source: Int J Mol Sci. 2025 Aug 28;26(17):8339. doi: 10.3390/ijms26178339 (PMC12428425; doi:10.3390/ijms26178339)

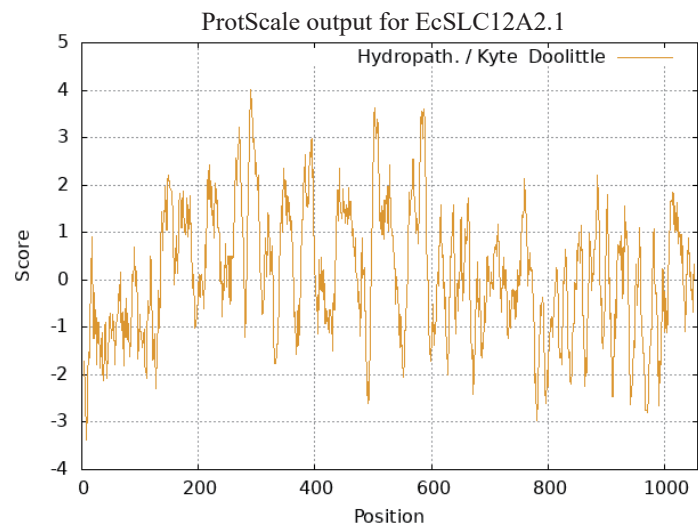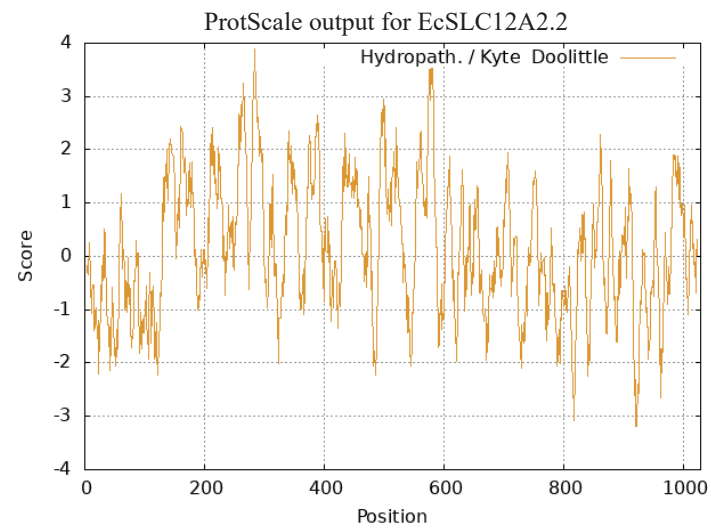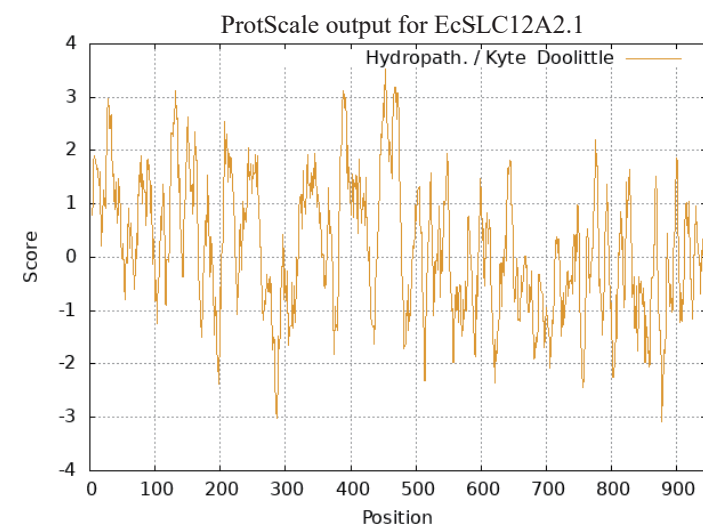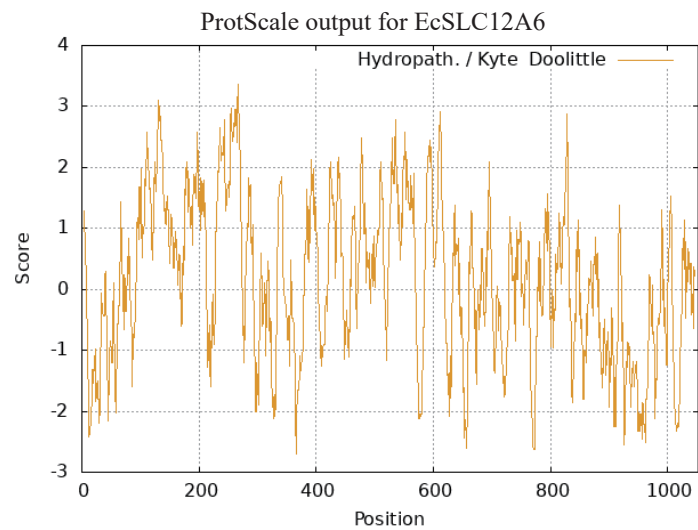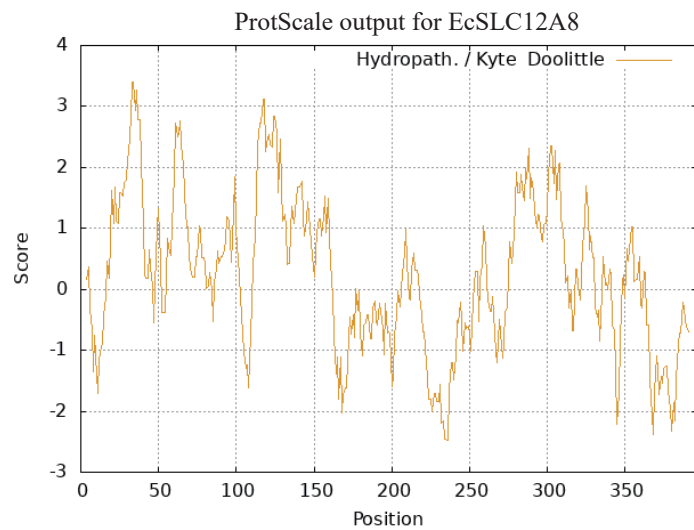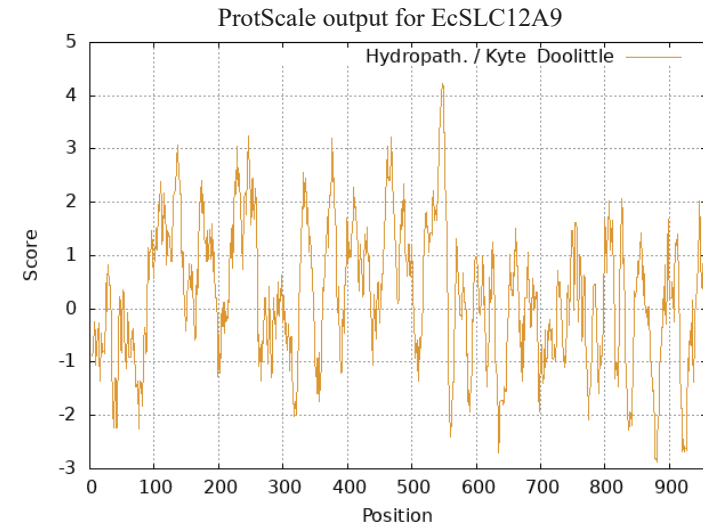

Supplement: Supplementary file 1 [file ijms-26-08339-s001.zip › Figure S1. Predicted hydrophilicity plot of the EcSLC12 protein..pdf]

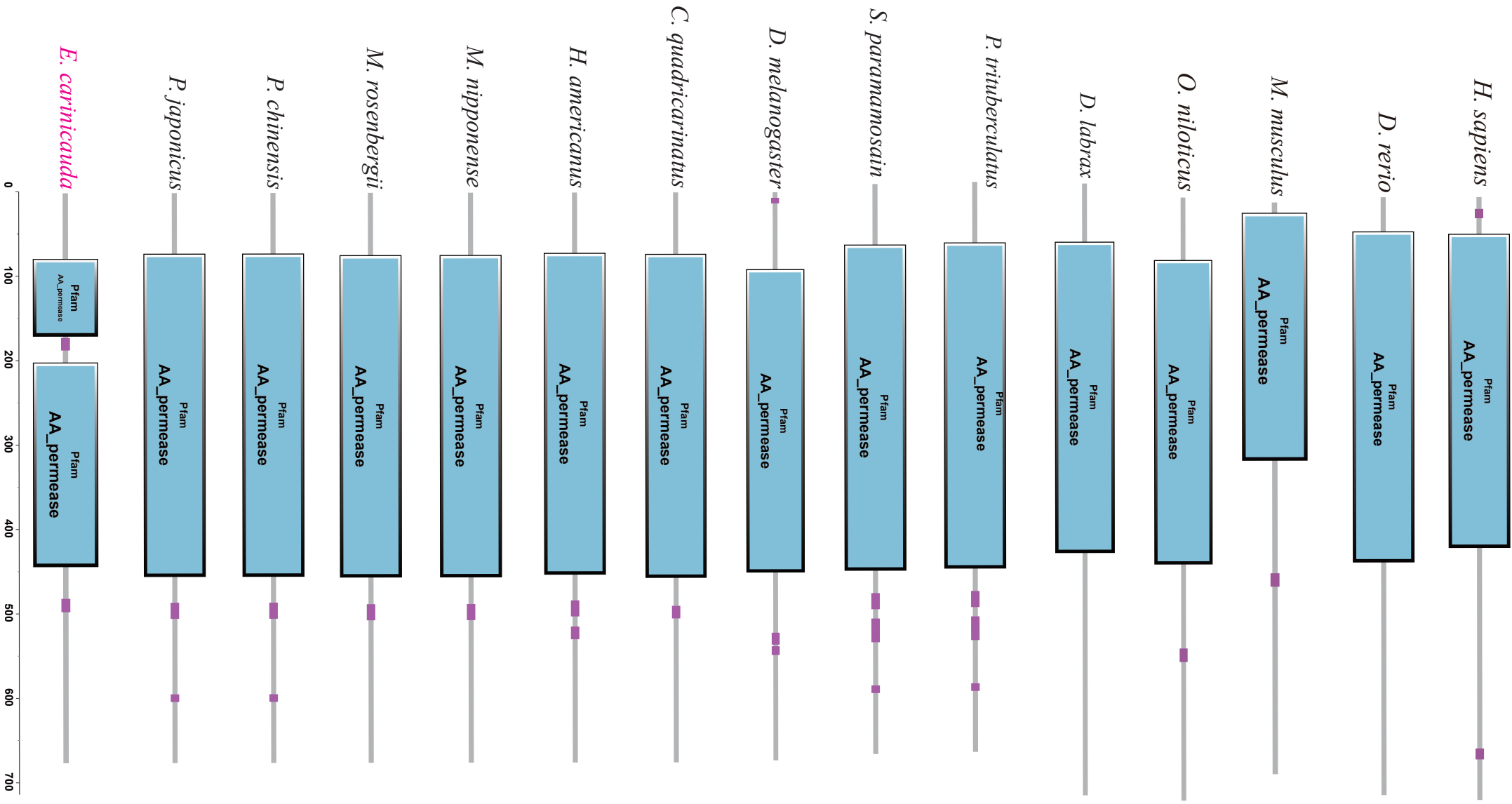

Supplement: Supplementary file 1 [file ijms-26-08339-s001.zip › Figure S2. Conserved domain analysis of SLC12A8 protein from 15 species. (2).pdf]
